# Dietary protein requirements of older adults with sarcopenia determined by the indicator amino acid oxidation technology

**DOI:** 10.3389/fnut.2025.1486482

**Published:** 2025-02-28

**Authors:** Wenxuan Wu, Fengge Chen, Hui Ma, Jiaxi Lu, Yanhong Zhang, Haisong Zhou, Yunqi Yang, Shuhui Nie, Rui Wang, Weixiao Yue, Min Li, Xiaoguang Yang

**Affiliations:** ^1^National Institute for Nutrition and Health, Chinese Center for Disease Control and Prevention, Beijing, China; ^2^Shijiazhuang Center for Disease Control and Prevention, Shijiazhuang, Hebei, China; ^3^Key Laboratory of Public Nutrition and Health, National Health Commission of the People's Republic of China, Beijing, China; ^4^Zhengding County Center for Disease Control and Prevention, Zhengding, Hebei, China

**Keywords:** protein requirement, older adults, sarcopenia, indicator amino acid oxidation, stable isotope

## Abstract

**Background:**

Although protein supplementation may be desirable in the management of sarcopenia, the appropriate protein requirements for older adults with sarcopenia are presently unclear.

**Objective:**

The aim of this study was to determine the protein requirements of older adults (65–81 year) with sarcopenia by using the indicator amino acid oxidation (IAAO) technology.

**Methods:**

Nine older adults with sarcopenia (four male participants and five female participants) participated in the study, with protein intakes ranging from 0.1 to 1.8 g/(kg·d). Each participant consumed an adapted diet with a protein level of 1.0 g/(kg·d) and 1.7 resting energy expenditure (REE) for 2 day. Day 3 was the oxidation day of the study. Diets that delivered energy at a 1.5 × REE were isocaloric. The amounts of phenylalanine and tyrosine maintained at a constant across intakes. Therefore, phenylalanine and tyrosine were added to the protein doses of 0.1–1.5 g/kg, which was based on the highest dose of lactalbumin content [1.8 g/(kg·d)]. Applying a non-linear mixed-effects model analysis of F^13^CO_2_, the protein requirement was determined by identifying the breakpoint in the F^13^CO_2_ data with graded amounts of dietary protein.

**Results:**

The mean estimated average requirement (EAR) and recommended nutrient intake (RNI) of protein for older adults with sarcopenia were 1.21 (95% CI: 0.95, 1.46) and 1.54 (95% CI: 1.13, 1.95) g/(kg·d), respectively.

**Conclusions:**

To our knowledge, this is the first study of protein intake in older adults with sarcopenia and indicates that older adults with sarcopenia may require a higher intake of dietary protein.

**Clinical trial registration:**

http://www.chictr.org.cn, ChiCTR2200061383.

## 1 Introduction

Sarcopenia is defined as an age-associated loss of skeletal muscle function and muscle mass that occurs in ~6%−22% of older adults, according to the International Clinical Practice Guidelines for Sarcopenia (ICFSR) (1). For older persons dwelling in the community, the prevalence of sarcopenia as defined by the European Working Group on Sarcopenia (EWGSOP) was 1%−29% (up to 30% in female) (2). The Asian Working Group for Sarcopenia (AWGS-2019) reported that the prevalence of sarcopenia in the Asian older population ranged from 5.5% to 25.7%, with male predominance (5.1%−21.0% in male and 4.1%−16.3% in female) (3). Sarcopenia is associated with increased risk of adverse outcomes, such as physical disability, depression, poor quality of life, hospital admissions, injurious falls, and death (4). Sarcopenia is recognized as a major public health issue (5).

Progress in developing effective drug treatments has been slow and there are currently no licensed drugs for sarcopenia (6). Adequate dietary protein intake is vital to maintaining muscle mass, as it ensures the availability of essential amino acids and stimulates protein synthesis (7). A meta-analysis has shown that older adults with sarcopenia consume significantly less protein than those without sarcopenia (8). Some studies suggest that higher protein intakes may help prevent age-related sarcopenia (9, 10). Therefore, protein requirements may be higher in older adults with sarcopenia. There is an urgent need to study the protein requirements of older people with sarcopenia.

The indicator amino acid oxidation (IAAO) technology has been used extensively to measure amino acid requirements and more recently to measure protein requirements of children (11), young males (12), pregnant females (13) and older adults (14–16). Even the protein requirements of children with phenylketonuria have recently been determined by the IAAO technique (17). And in our previous study, we conducted protein requirements of older Chinese adults using IAAO technology (18). The research that are currently available on the protein requirements of older adults are based on healthy older adults; however, these studies do not address the protein requirements of older people with sarcopenia. We subsequently reassessed the protein requirements of healthy older adults in China by excluding sarcopenia (19), but data on the protein requirements of older adults with sarcopenia were still lacking. Therefore, the objective of this study was to determine dietary protein requirements of older people with sarcopenia by using the IAAO technique, with L-[1-^13^C] phenylalanine as the indicator amino acid, in order to improve nutritional therapy in this population and promote healthy aging.

## 2 Materials and methods

### 2.1 Subjects

Twelve older adult volunteers (5 males and 7 females) were recruited in the study which began in August 2022, after underwent a diagnosis of sarcopenia and a routine medical examination, including complete blood count, blood chemistry, and hepatic and renal functions. The inclusion criteria of subjects included were age 65–81 year; no weight loss in the previous 6 month; no chronic disease or acute illness known to influence protein and amino acid metabolism such as diabetes, kidney disease, liver disease or cancer; no gastrointestinal diseases; and no recent infectious diseases, surgery, or antibiotic therapy within 8 week of the commencement of this study. Participants with hypertension were not excluded if their blood pressure was well controlled and their antihypertensive medications were taken as prescribed by their physician. Due to the need to collect exhalation, older age with respiratory diseases were also excluded. Participants had sarcopenia, who were diagnosis by the 2019 AWGS diagnostic criteria (3). The detailed progression of study inclusion and exclusion is shown in Supplementary Figure S1. The present study was conducted according to the guidelines in the Declaration of Helsinki and was approved by the Ethical Committee of the Institute for Nutrition and Health, Chinese Center for Disease Control and Prevention (No. 2022-021). Each subject provided signed informed consent prior to the initiation of the study. This trial was registered at the Chinese clinical trial registry as ChiCTR2200061383.

### 2.2 Diagnosis of sarcopenia

Several international sarcopenia working groups have published expert consensus and diagnostic criteria, and the diagnostic criteria are constantly being updated and improved (3, 20–22). This study employed the AWGS 2019 updated diagnostic criteria. Sarcopenia is diagnosed when low muscle mass plus low muscle strength or low physical performance are measured. When low muscle strength, low muscle mass and low physical performance are measured, it will be considered as severe sarcopenia. Muscle mass was assessed by measuring appendicular skeletal muscle mass index (ASMI, kg/m^2^) which was calculated by appendicular skeletal muscle mass (ASM) divided by height squared (m^2^). ASM was calculated by the sum of the skeletal muscle mass of the upper and lower limbs using bioelectrical impedance analysis (BIA) (Inbody 720, Biospace Co. Ltd., Seoul, Korea). Participants should remove metal items such as necklaces and watches before starting to measure ASM. The definition of low muscle mass was ASMI < 7.0 kg/m^2^ for males and < 5.7 kg/m^2^ for females. Muscle strength was assessed by measuring the power of handgrip strength (HGS) using the hand dynamometer. The cut-off points for low HGS were < 18 kg for female and < 28 kg for male. Six-meter gait speed (GS) or 5-time chair stand test (CS-5) assessed physical performance. Low physical performance was defined as a GS < 1 m/s or CS-5 ≥ 12 s.

### 2.3 Experimental design

The experimental design was based on the minimally invasive IAAO protocol used previously in healthy older adults to determine protein requirements (14, 18). Before the studies commenced, each participant was fasted overnight (12 h) and body composition [fat-free mass (FFM) and ASM] and REE were measured. Body composition was measured by BIA. REE was measured by continuous, open-circuit indirect calorimetry (Cosmed K5, COSMED Srl, Rome, Italy). Two days before the study day were the adaptation days, during which the participants received a daily protein intake of 1.0 g/kg body weight and 1.7 × REE. The adaptation diet was weighted in daily portions for each subject. The diet was a standard Chinese diet, mostly consisting of high-protein meals and staple foods. The daily 1.0 g/(kg·d) protein intake was calculated for each participant according to body weight. Each individual food item in the meal was prepared separately. During the adaptation days, all participants were encouraged to consume all the food provided. Food consumption of participants was recorded using a diet record sheet. The data collection encompassed both the type and quantity of food consumed by all participants, with detection of macronutrient concentrations (including protein, fat, and carbohydrate) as well as energy content to ensure that participants' actual protein intakes closely approximated 1.0 g/(kg·d). Participants were also provided a multivitamin and mineral tablet (Centrum; Wyeth Consumer Health Care) supplement daily for the duration of all studies.

On the study day, after a 12-h fast, participants were completely randomized to consume 1 of 7 dietary protein intakes [0.1, 0.3, 0.6, 0.9, 1.2, 1.5, and 1.8 g/(kg·d)], with a 1-week period between doses. All participants consumed 8 hourly isocaloric meals, and the energy was provided at 1.5 × REE. The experimental diet consisted of the following: lactalbumin powder, protein-free biscuits, protein-free and fried starch slices, and protein-free lotus root starch. To keep the diets isocaloric, the carbohydrate content of the diets was adjusted according to the amount of protein intake. The study diet provided 33% energy as fat and variable energy from carbohydrate (42.6%−65.4%) and protein (1.0%−21.7%) according to the test protein intake. Each protein dose provided the same amounts of phenylalanine and tyrosine, which was ensured by determination of the amino acid composition of lactalbumin powder according to the Chinese standard GB 5009.124-2016. L-phenylalanine (Now Foods) and L-tyrosine (Puritan's Pride) were added to protein doses of 0.1–1.5 g/kg according to the highest dose of daily protein content (1.8 g/kg). The amount of L-[1-^13^C] phenylalanine given during the study day was subtracted from the dietary provision of phenylalanine such that the total phenylalanine intake was 54.4 mg/(kg·d). Tyrosine was provided at 58.0 mg/(kg·d) to ensure an excess of tyrosine (23). All participants completed the study within 3-month. Amino acid compositions of selected test protein intakes are provided in Table 1.

**Table 1:** Amino acid composition of reference protein and various test protein doses.

	**Reference protein^a^, mg/g**	**0.1 g protein/kg, mg/0.1 g**	**0.3 g protein/kg, mg/0.3 g**	**0.6 g protein/kg, mg/0.6 g**	**0.9 g protein/kg, mg/0.9 g**	**1.2 g protein/kg, mg/1.2 g**	**1.5 g protein/kg, mg/1.5 g**	**1.8 g protein/kg, mg/1.8 g**
L-Alanine	53.8	5.4	16.1	32.3	48.4	64.6	80.7	96.8
L-Arginine	23.3	2.3	7.0	14.0	21.0	28.0	35.0	41.9
L-Aspartic acid	106.9	10.7	32.1	64.1	96.2	128.3	160.4	192.4
L-Cysteine	11.3	1.1	3.4	6.8	10.2	13.6	17.0	20.3
L-Glutamic acid	182.4	18.2	54.7	109.4	164.2	218.9	273.6	328.3
L-Glycine	18	1.8	5.4	10.8	16.2	21.6	27.0	32.4
L-Histidine	17.4	1.7	5.2	10.4	15.7	20.9	26.1	31.3
L-Isoleucine	62.8	6.3	18.8	37.7	56.5	75.4	94.2	113.0
L-Leucine	103.9	10.4	31.2	62.3	93.5	124.7	155.9	187.0
L-Lysine	90.1	9.0	27.0	54.1	81.1	108.1	135.2	162.2
L-Methionine	22.9	2.3	6.9	13.7	20.6	27.5	34.4	41.2
L-Valine	59.4	5.9	17.8	35.6	53.5	71.3	89.1	106.9
L-Proline	45.9	4.6	13.8	27.5	41.3	55.1	68.9	82.6
L-Serine	51.1	5.1	15.3	30.7	46.0	61.3	76.7	92.0
L-Threonine	75.1	7.5	22.5	45.1	67.6	90.1	112.7	135.2
L-Tryptophan	15.2	1.5	4.6	9.1	13.7	18.2	22.8	27.4
L-Phenylalanine^b^	30.2	3.0	9.1	18.1	27.2	36.2	45.3	54.4
Added L-phenylalanine^b^	—	51.3	45.3	36.2	27.2	18.1	9.1	0.0
L-Tyrosine^c^	32.2	3.2	9.7	19.3	29.0	38.6	48.3	58.0
Added L-tyrosine^c^	—	54.7	48.3	38.6	29.0	19.3	9.7	0.0

a Represents lactalbumin composition.

b L-Phenylalanine intake was kept constant at 54.4 mg/(kg·d).

c L-Tyrosine intake was kept constant at 58.0 mg/(kg·d).

### 2.4 Tracer protocol

**Figure 1:**
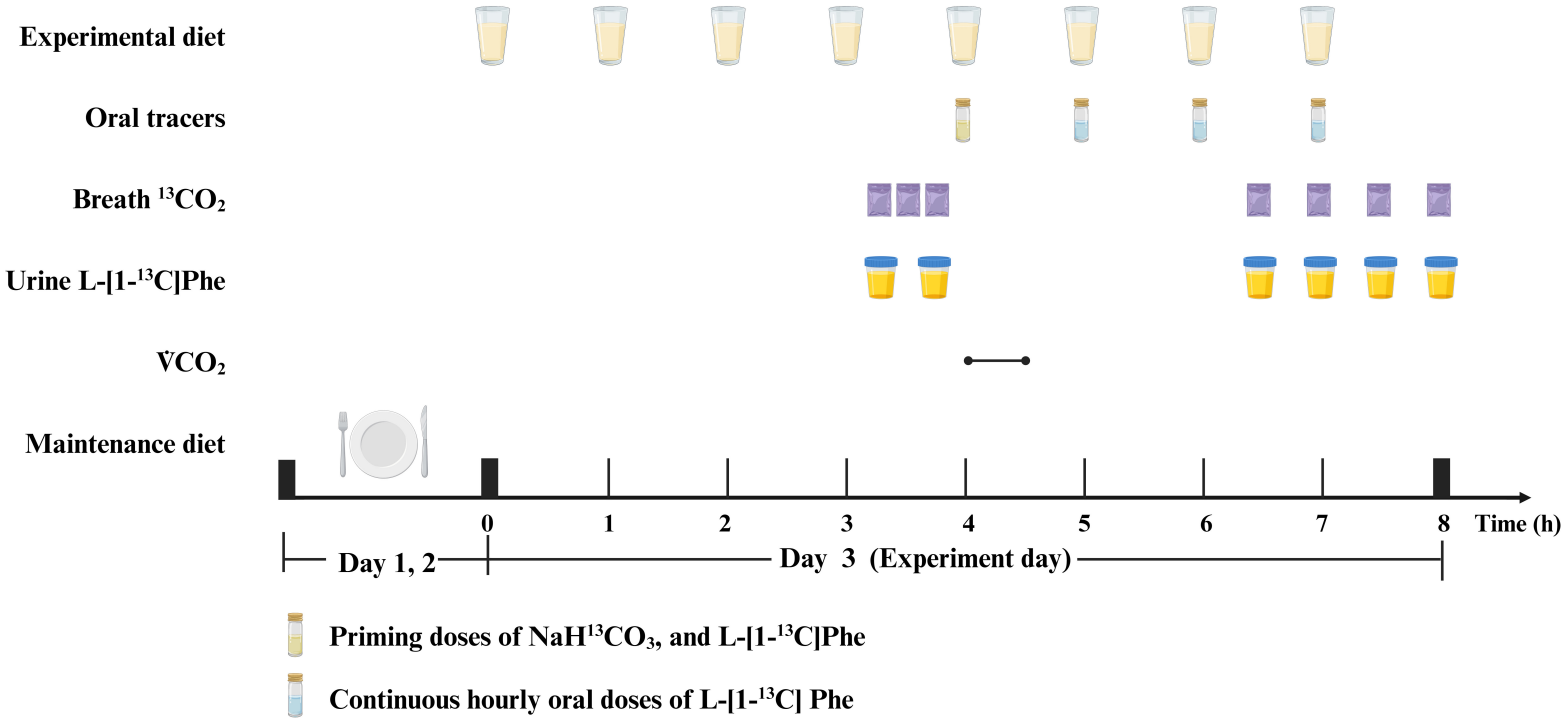
Example protocol for an indicator amino acid oxidation (IAAO) study. Created with BioRender.com.

At each protein dose on each oxidation study day, the participants consumed hourly meals for 4 h prior to the start of the oral tracer infusion protocol. Oral priming doses of 0.176 mg NaH^13^CO_3_/kg (99 atom percentage excess; Cambridge Isotope Laboratories) and 0.66 mg L-[1-^13^C] phenylalanine/kg (99 atom percentage excess; Cambridge Isotope Laboratories) were provided at the fifth hourly meal. An hourly oral dose of 1.2 mg/(kg·h) of L-[1-^13^C] phenylalanine was commenced simultaneously with the fifth meal and continued for the remaining 3 h of the study. A detailed protocol is provided in Figure 1.

### 2.5 Sample collection and analysis

Breath and urine samples were collected on all oxidation study days. Before the tracer protocol began, three baseline breath samples (45, 30, and 15 min before) and two baseline urine samples (45 and 15 min before) were collected. After the tracer protocol began, four plateau breath and four plateau urine samples were collected at isotopic steady state every 30 min beginning 2.5 h.

Breath samples were obtained and stored at room temperature for further analysis in disposable expiratory bags (Shenzhen Zhonghe Headway Bio-Sci & Tech Co., Ltd). The enrichment of ^13^CO_2_ in breath samples was analyzed using a ^13^C-breath test device (HeliFANplus, Fischer ANalysen Instrumente GmbH). Prior to analysis, urine samples were kept at −80°C. Using a TSQ triple quadrupole mass spectrometer (Thermo Fisher Scientific) in positive electrospray ionization mode, as described previously (24), the enrichment of L-[1-^13^C] phenylalanine in urine samples was measured. Isotopic enrichment was expressed as mole percentage excess and was calculated from peak area ratios at the isotopic steady state at baseline and at plateau.

### 2.6 Tracer kinetics

Isotope kinetics were calculated as previously described (14, 18, 25). The ^13^CO_2_ rate of appearance in breath [F^13^CO_2_, μmol/(kg·h)] after the oxidation of ingested L-[1-^13^C] phenylalanine was calculated by using a factor of 0.82 to account for carbon dioxide retained in the body's bicarbonate pool.

It was previously shown that plasma amino acid enrichments can be determined from urine (26–28). Whole-body phenylalanine flux [μmol/(kg·h)] was calculated from the dilution of orally administered L-[1-^13^C] phenylalanine into the plasma pool (at isotopic steady state) by using enrichment of L-[1-^13^C] phenylalanine in urine.

### 2.7 Statistical analysis

Statistical analyses were performed by using the Statistical Analysis Systems statistical software (version 9.4; SAS Institute Inc., Cary, NC). All of the results are expressed as means ± SDs. Differences were considered significant at *P* < 0.05.

ANOVA was used to test for differences in the body weight, FFM, ASMI and phenylalanine flux and if there was a general difference, a *post hoc* analysis was performed by using the Student Newman–Keuls test for multiple comparisons. Student's *t*-test was used to determine differences between mean protein requirements of older adults with sarcopenia in the current study and older adults without sarcopenia from our previous study (19). The mean protein requirement was estimated by applying a non-linear mixed-effects model (PROC NLMIXED, SAS Institute) to the F^13^CO_2_ data as previously described (14, 18).

The SAS Procedure NLMIXED used in our analysis is able to provide estimates for arbitrary functions of the parameters with CIs. Using this approach, we obtained an estimate with 95% CI for the EAR. CIs were obtained by following the standard asymptotic theory of the maximal likelihood estimation, but the method narrowed the 95% CIs (29). Therefore, to determine RNI, we further combined SD (EAR plus 1.96 SD) (23, 30).

## 3 Results

### 3.1 Subject characteristics

Twelve participants with sarcopenia were recruited to participate. However, three participants withdrew from the study due to personal issues. We adopted the AWGS 2019 recommended diagnostic algorithm (3), of which five were diagnosed with sarcopenia and four with severe sarcopenia. ASM measurements were performed weekly to better monitor the subject's muscle mass. Subject characteristics are presented in Table 2. Their ages ranged from 66 to 81 year, and BMIs (in kg/m^2^) ranged from 18.5 to 25.3. Each subject was randomly assigned to receive a concentration of 0.1, 0.3, 0.6, 0.9, 1.2, 1.5, and 1.8 g protein/kg body weight. Six participated in 7 separate study days, and three participated in 5 study days; thus, nine older people were studied for a total of 57 isotope tracer IAAO studies. There were no significant differences in body weights, ASMI and FFM of subjects at different doses of dietary protein in Supplementary Table S1.

**Table 2:** Subject characteristics of older adults with sarcopenia.^a^

**Variable**	**Subject sex**	
	**Male (*n* = 4)**	**Female (*n* = 5)**
Age, year	73.3 ± 5.9	75.4 ± 4.6
Weight, kg	55.1 ± 6.0	49.6 ± 4.9
Height, cm	164.4 ± 5.7	150.8 ± 7.4
BMI, kg/m^2^	20.3 ± 1.2	21.9 ± 2.6
REE, kcal/d	1290 ± 79	1180 ± 79
FFM, kg	42.6 ± 4.5	32.0 ± 2.0
%Fat	23.6 ± 4.2	35.8 ± 7.4
**Diagnosis of sarcopenia**		
Handgrip strength, kg	27.5 ± 2.8	12.2 ± 2.2
6-m walk, m/s	1.2 ± 0.1	1.1 ± 0.1
5-time chair stand test, s	12.0 ± 5.1	14.2 ± 3.2
ASMI, kg/m^2^	6.4 ± 0.4	5.2 ± 0.3

a Results are expressed as means ± SDs. ASMI, appendicular skeletal muscle mass index; FFM, fat-free mass; REE, resting energy expenditure; %Fat, percentage of body fat.

### 3.2 Dietary nutrient intakes

The diets with different protein doses are consistent, and the designed protein doses were maintained at 1.0 g/(kg·d) during the 2 days before the tracer protocol. The actual main macronutrient and energy intakes of the subjects for the 7 dietary protein doses were 1.05 ± 0.09 g/(kg·d) for protein, 0.92 ± 0.10 g/(kg·d) for fat, 5.58 ± 0.59 g/(kg·d) for carbohydrate, and 35.09 ± 3.46 kcal/(kg·d) for energy.

### 3.3 Breath ^13^CO_2_ excretion

**Figure 2:**
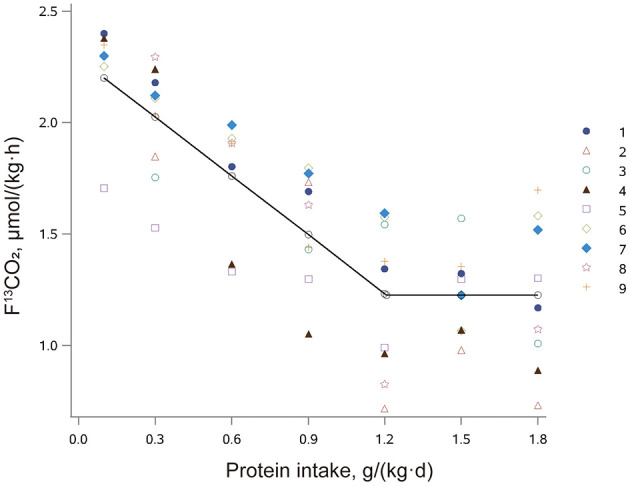
The relation between protein intake and breath F^13^CO_2_ production in older Chinese adults with sarcopenia given seven different doses of protein. The data points represent the ^13^CO_2_ excretion of individual subjects at each protein intake dose. The breakpoint represents the estimated mean protein requirement, which is 1.21 g/(kg·d).

The influence of protein intake on production of ^13^CO_2_ from phenylalanine oxidation (F^13^CO_2_) in older adults with sarcopenia is shown in Figure 2. F^13^CO_2_ decreased as protein intake increased, consistent with an increase of L-[1-^13^C] phenylalanine incorporation into the protein. This increase in incorporation of labeled phenylalanine continued until the protein intake reached the protein requirement and there was no further incorporation of labeled phenylalanine into the protein. Nonlinear mixed-effects model analysis of the F^13^CO_2_ data resulted in the identification of a breakpoint for the mean protein requirement of 1.21 (95% CI: 0.95, 1.46) g/(kg·d). The RNI (EAR plus 1.96 SD) was 1.54 (95% CI: 1.13, 1.95) g/(kg·d).

### 3.4 Phenylalanine flux

Phenylalanine flux was not affected (*P* = 0.6148) within each individual by differences in protein intake as required by the IAAO technique. The phenylalanine flux was 25.6 ± 6.6 μmol/(kg·h). Thus, the precursor pool of phenylalanine was consistent across trials, which is a requirement for the reliable estimation of the recommended protein intake by F^13^CO_2_ analysis.

### 3.5 Comparison of requirements between older adults with sarcopenia (current study) and older adults without sarcopenia

Table 3 compares the protein requirement estimate and metabolic data of the older adults with sarcopenia (current study) with that of healthy older people in our previous study (19). There was no significant difference in %Fat, 6-meter walk, 5-time chair stand test or phenylalanine flux. Older adults with sarcopenia in this study weighed less than older adults without sarcopenia in our previous study (52.1 ± 5.8 kg compared with 65.9 ± 8.1 kg; *P* < 0.01). In our current study, six older adults with sarcopenia exhibited low grip strength, two had low 6-m walk, and six had low 5-time chair stand test. In our previous study, three older adults without sarcopenia exhibited low 5-time chair stand test results but normal ASMI in the extremities. As expected, BMI, REE, FFM, and ASMI were significantly lower in older adults with sarcopenia than did older adults without sarcopenia (*P* < 0.01). There was no statistically difference in EAR between the two groups when expressed per kilogram of body weight. However, the protein requirement per kilogram of FFM was significantly higher in older adults with sarcopenia than in older adults without sarcopenia (*P* = 0.0018).

**Table 3:** Comparison of requirements between older adults with sarcopenia (current study) and older adults without sarcopenia.^a^

**Variable**	**Sarcopenia**	**Health^b^**	***P***
Age, year	74.4 ± 5.0	70.8 ± 3.4	0.0376
*n*	9	16	
Weight, kg	52.1 ± 5.8	65.9 ± 8.1	0.0002
BMI, kg/m^2^	21.2 ± 2.2	25.8 ± 2.4	<0.0001
REE, kcal/d	1229 ± 94	1514 ± 171	<0.0001
FFM, kg	36.7 ± 6.4	45.3 ± 6.7	0.005
%Fat	30.4 ± 8.7	32.6 ± 5.7	NS
Handgrip strength, kg	19.0 ± 8.4	26.4 ± 6.8	0.0241
6-m walk, m/s	1.1 ± 0.1	1.2 ± 0.1	NS
5-time chair stand test, s	13.2 ± 4.1	11.2 ± 2.3	NS
ASMI, kg/m^2^	5.7 ± 0.7	7.2 ± 0.8	0.0002
Phenylalanine flux, μmol/(kg·h)	25.6 ± 6.6	25.8 ± 8.1	NS
EAR_BW_, g/(kg·d)	1.21	0.94	NS
RNI_FFM_, g/(kg·d)	1.74	1.38	0.0018

a Results are expressed as means ± SDs. Comparisons between older adults with sarcopenia in the current study and older adults without sarcopenia were performed by *t* test. Differences were considered significant at *P* < 0.05. ASMI, appendicular skeletal muscle mass index; EAR, estimated average requirement; FFM, fat-free mass; REE, resting energy expenditure; RNI, recommended nutrient intake; %Fat, percentage of body fat.

b Data for older adults are from (19).

## 4 Discussion

The objective of this study was to investigate the estimated EAR and RNI in Chinese older people with sarcopenia utilizing, for the first time in this population, the minimally-invasive IAAO technique. Our data revealed that the protein EAR and RNI of older people with sarcopenia to be 1.21 and 1.54 g/(kg·d), respectively. The protein EAR obtained in this study for older adults with sarcopenia does not differ from the estimates in older adults without sarcopenia (19). But on the basis of FFM, the current estimated protein requirement for older adults with sarcopenia was 1.74 g/(kg·d), which was significantly higher compared with the estimated protein requirement for older adults without sarcopenia of 1.38 g/(kg·d) (19). The results of this study suggest that older adults with sarcopenia may have higher protein requirements than those without sarcopenia.

Multiple factors are involved in the process of muscle mass loss (31), of which nutrition, especially protein intake, is one of the most important and modifiable factors (7). Protein ingestion stimulates muscle protein synthesis rates and suppresses, likely via insulin, muscle protein breakdown rates (32). To preserve muscle mass, adequate amounts of dietary protein should be consumed (31, 33).

In vulnerable populations such as older adults, the minimally invasive IAAO technique is more practical and less invasive than nitrogen balancing in determining protein requirements (30). In our previous study in 16 Chinese older adults (>65 year) without sarcopenia that used the IAAO technique, the protein EAR and RNI were determined to be 0.94 and 1.36 g/(kg·d), respectively (19). In the present study, the protein requirements of older adults with sarcopenia were about 30% higher than those of the studies mentioned above (14–16, 18).

In recent years, some studies recommend that older people should consume 1.2–2.0 g/(kg·d) or more of protein, depending on their health status (34, 35). Evidence suggests that higher dietary protein intake is beneficial to older adults in keeping good health, promoting disease recovery and maintaining function (36). In a cross-sectional examination of 3,213 community-based middle-aged and older persons (mean age: 60.7 years) in China, it was shown that participants who consumed ≥ 1.68 g/(kg·d) had the lowest risk of having low muscle mass (37). A 12-week, double-blind, randomized, controlled study in undernourished prefrail and frail older subjects aged 70–85 years showed that protein intake of 1.5 g/(kg·d) had the most beneficial effect in preventing sarcopenia and frailty compared with protein intakes of 0.8 and 1.2 g/(kg·d) (38). These results also support that older adults with sarcopenia need to consume more protein daily. Most older adults with acute or chronic illnesses require 1.2–1.5 g/(kg·d) of dietary protein; those with serious illness or injury or severely malnutrition may require up to 2.0 g/(kg·d) (36, 39). Although potential negative consequences of a higher protein have been suggested, namely that a diet higher in protein could lead to renal injury and affect bone health, there is currently no scientific foundation for this in human studies (10, 40, 41). However, for patients with sarcopenia combined with renal disease, a reasonable protein intake should be set under the guidance of a clinician, and health education and malnutrition monitoring should be implemented.

The approximation for the RNI can also be calculated from the coefficient of variation (CV) of the protein EAR as recommended in the Institution of Medicine report (23). In the present study, the approximate of the RNI using 1.25 times the EAR is 1.51 g/(kg·d). A recent study estimated the inter-individual variability as a CV of ~20% based on seven IAAO studies (42). The CV of the protein requirements determined by IAAO technique was wider than the ordinary CV obtained from the nitrogen balance test. The CV-weighted mean of three studies conducted at steady state, among adults or older age without exercise load, was 19.0% (42). The use of this CV (19.0%) to infer the protein RNI resulted in the RNI of 1.67 g/(kg·d), which is higher than the 1.54 g/(kg·d) obtained by combining standard deviations. These studies are limited and individual differences in older adults may be large, which requires further data to determine whether a CV of 19% is appropriate.

In this study, we have attempted to remove the F^13^CO_2_ values at the test protein dose of 0.1 g/(kg·d) and found essentially no difference in the results from when they were not removed. If a test protein dose such as 2.0 g/(kg·d) is added after the 1.8 g/(kg·d) protein dose, the model fit may be even better. Therefore, we recommend that a test protein dose of 0.3–2.0 g/(kg·d) would be more appropriate for older people with sarcopenia. Apart from this, there was no statistically difference in EAR per kilogram of body weight between the two groups. This lack of statistically differences may be attributed to the insufficient sample size (one of the limitations of this study) and the lower body weight of the older adults with sarcopenia. Further studies are warranted to differentiate between genders and determine the protein requirement in older adults with sarcopenia.

The IAAO technology is controversial (43–45), and has been criticized on methodologic grounds (45). The main issues include the selection and intake of the indicated amino acids, and the length of the adaptation period. The criticisms were discussed in the response of Scherbinsky et al. as well as in recent studies (44, 46, 47). The design of the IAAO studies requires a range of test amino acid or protein intakes be studied, holding the indicator amino acid intake constant (30). Thus, any changes in IAAO are reflective of changes in the test amino acid or protein intakes and inversely associated with protein synthesis. In different protein level diets, the addition of phenylalanine and tyrosine was made to ensure their intakes matched their content in the highest protein level, and this meant that phenylalanine and tyrosine was never limiting for the utilization of the protein in the meals. This was in contrast to most of the other studies where the IAAO technique was used to study protein requirements where the much lower intakes of phenylalanine and tyrosine might have limited protein utilization at high levels of protein intake (45).

In conclusion, our results suggest that the mean EAR of protein in older Chinese adults with sarcopenia is 1.21 (95% CI: 0.95, 1.46) g/(kg·d), and the RNI is 1.54 (95% CI: 1.13, 1.95) g/(kg·d). Older adults with sarcopenia may need higher protein intake than Chinese older adults without sarcopenia. Due to the small sample size in this study as well as the variation between and within-subject, more studies with larger sample sizes and repeated measures trials are needed to evaluate the protein requirements of older people with sarcopenia.

## Data Availability

The raw data supporting the conclusions of this article will be made available by the authors, without undue reservation.
