# Supplementary material for: Dietary protein requirements of older adults with sarcopenia determined by the indicator amino acid oxidation technology
Source: Front Nutr. 2025 Feb 28;12:1486482. doi: 10.3389/fnut.2025.1486482 (PMC11906324; doi:10.3389/fnut.2025.1486482)
Supplement: Supplementary file 1 [file Table_1.docx]

Supplementary Material

# Supplementary Figures and Tables

**Supplementary Table 1.** Body weight of older Chinese adults with sarcopenia administered different protein doses in 7 wk

|  | 1 wk | 2 wk | 3 wk | 4 wk | 5 wk | 6 wk | 7 wk |
| --- | --- | --- | --- | --- | --- | --- | --- |
| **Body weight, kg** |  |  |  |  |  |  |  |
| Male | 55.2 ± 6.0 | 55.1 ± 5.9 | 55.4 ± 5.9 | 54.3 ± 6.6 | 55.9 ± 5.5 | 55.7 ± 5.4 | 55.6 ± 6.7 |
| Female | 49.1 ± 4.2 | 49.2 ± 4.1 | 49.4 ± 4.4 | 49.2 ± 4.7 | 49.9 ± 4.8 | 49.8 ± 4.7 | 50.5 ± 5.1 |
| All | 52.9 ± 5.8 | 52.9 ± 5.6 | 52.4 ± 5.7 | 51.4 ± 5.8 | 52.8 ± 5.8 | 52.6 ± 5.7 | 52.4 ± 5.9 |
| **ASMI, kg/m^2^** |  |  |  |  |  |  |  |
| Male | 6.4 ± 0.4 | 6.3 ± 0.4 | 6.3 ± 0.2 | 6.3 ± 0.6 | 6.3 ± 0.2 | 6.2 ± 0.3 | 6.3 ± 0.3 |
| Female | 5.5 ± 0.1 | 5.5 ± 0.1 | 5.1 ± 0.3 | 5.2 ± 0.4 | 5.2 ± 0.4 | 5.1 ± 0.4 | 5.2 ± 0.5 |
| All | 6.0 ± 0.6 | 5.9 ± 0.5 | 5.7 ± 0.7 | 5.6 ± 0.7 | 5.7 ± 0.7 | 5.6 ± 0.7 | 5.6 ± 0.7 |
| **FFM, kg** |  |  |  |  |  |  |  |
| Male | 42.6 ± 4.5 | 42.4 ± 4.3 | 42.6 ± 3.1 | 43.2 ± 5.5 | 42.5 ± 3.1 | 42.2 ± 4.2 | 42.7 ± 4.5 |
| Female | 33.0 ± 1.7 | 33.3 ± 2.0 | 32.2 ± 2.2 | 31.9 ± 2.4 | 32.3 ± 2.7 | 31.8 ± 1.8 | 32.4 ± 2.3 |
| All | 38.5 ± 6.1 | 38.5 ± 5.9 | 36.8 ± 6.0 | 36.1 ± 6.8 | 36.8 ± 6.0 | 36.4 ± 6.2 | 36.8 ± 6.3 |

## ^1^Results are expressed as means ± SDs, *n* = 9. By ANOVA, there were no significant differences in subject body weights, ASMI and FFM, *P* >0.05. ASMI, appendicular skeletal muscle mass index; FFM, fat-free mass.Supplementary Figures


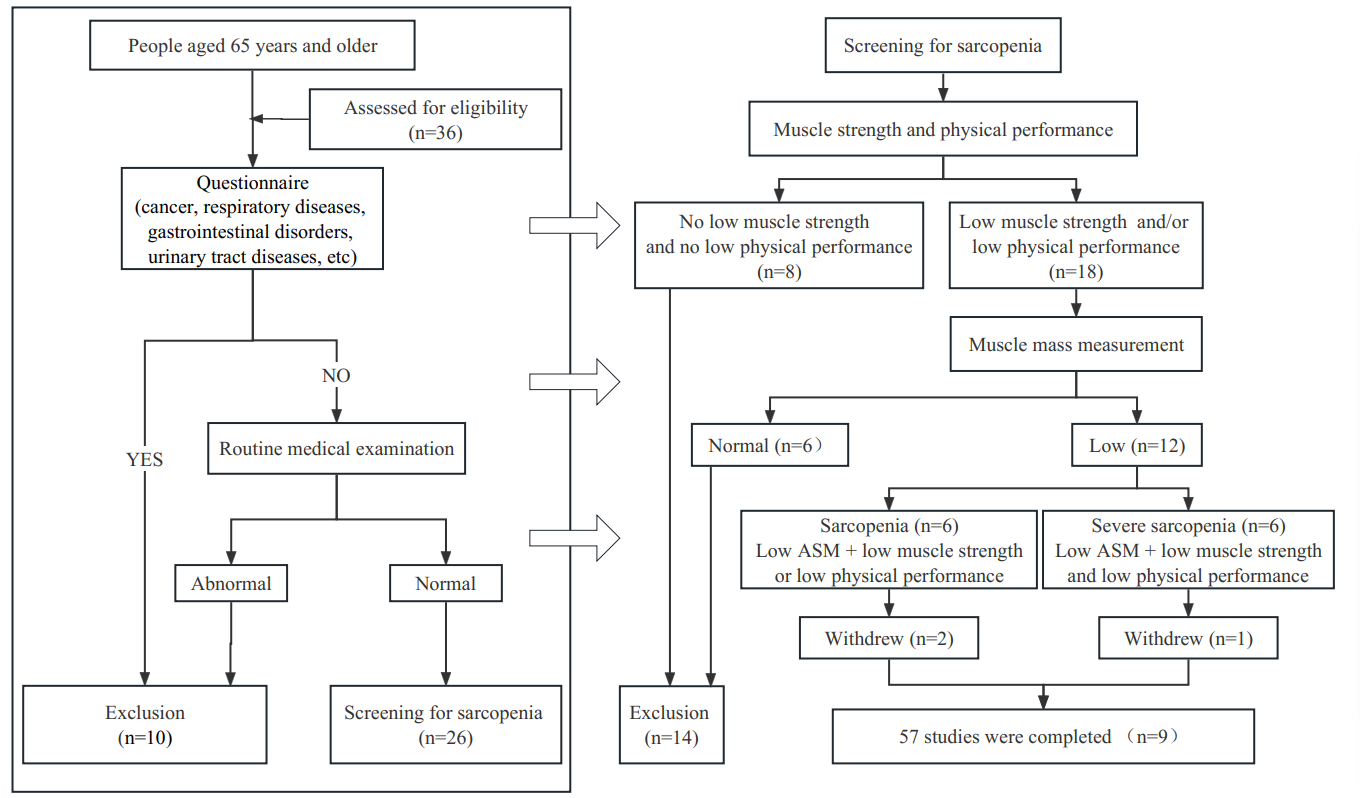


**Supplementary Figure 1.** Diagram of the screening method for sarcopenia according to the 2019 Asian Working Group for Sarcopenia (AWGS) criteria and the number of participants at each step.
